# Range-Programming Stripping Voltammetry for Determination of Some Metals in Seawater

**DOI:** 10.6028/jres.093.051

**Published:** 1988-06-01

**Authors:** Myung-Zoon Czae, Jong-Hyup Lee

**Affiliations:** Department of Chemistry, Hanyang University, Seoul 133, Korea

Multielement capability of stripping analysis is one of its distinctive advantages described elsewhere [1]. Anodic stripping voltammetry with the use of differential pulse mode (DPASV) is uniquely suited [2], and therefore, applied extensively to the direct simultaneous determination of some trace metals (Zn, Cd, Pb, and Cu) in sea waters [3,4]. Even with the well-established procedure for a given sample, there still arises the problem introduced by simultaneously measuring relative concentrations of metal ions. A case in point is zinc and cadmium concentrations whose ratio sometimes is as high as 400 (12 ppb Zn/0.03 ppb Cd), and typically as high as 200 in raw surface sea waters [3]. In such a case, either one must run the entire steps separately by selecting suitable variables for each metal or pair of metals whose concentration ratio is adequate for simultaneous measurement, or the recorder scale must be changed during a single run to obtain the best results. The latter approach, difficult to achieve manually in practice, would rely on the application of an autoranging amplifier [5]. The use of an autoranging amplifier, however, has given rise to complications in evaluating the resulting voltammograms in most practical applications for analyzing seawaters [6]. The present work was initiated in order to overcome deterioration in readability and data quality, by developing a new programmed-ranging technique.

## Principle of Operation

**Figure 1:**
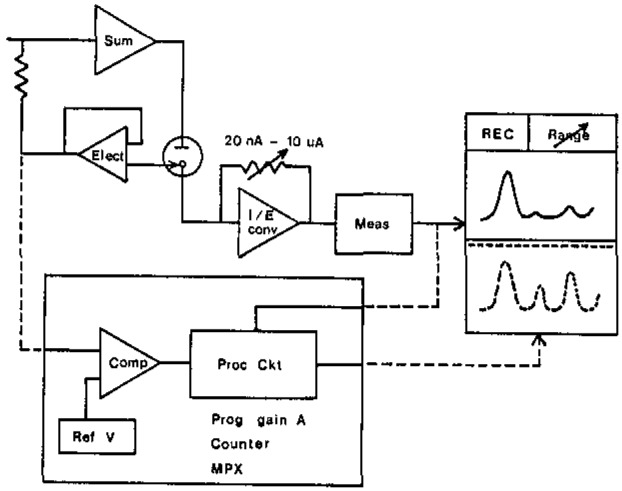
Schematic diagram of proposed technique.

With the pulsed (commonly DP) stripping mode in the simultaneous measurements, each voltammogram peak starts from the baseline of nearly zero or very small current when moderate resolution is provided. This fact enables us to change current gain to a pre-specified value (programmed vs potential, according to the concentration ratios for a lot of samples), when the potential is scanned to a position just before the start of a subsequent peak (where pre-peak current also drops nearly to the baseline). Figure 1 illustrates the basic principle behind the programmed-ranging technique. If we incorporate an additional amplifier (fig. 1, lower large box) with a gain (programmable) of *G_j_*(*x*) to a prototyping polarographic analyzer’s output (which otherwise would give the peak current *i_j_*, for the *j*th component or peak), the overall analytical signal (*I_j_*) will be

$$I_{j}=G_{j}\left(x\right)\;i_{j}$$ (1)

where gain *G_j_*(*x*) depends on some variable *x*. In a scheme employing an autoranging amplifier, *x* is *I_j_* provided by feedback means. Thus, gain *G_j_*(*I_j_*) varies indefinitely in a stepwise manner by a predetermined factor of (10)^1/2^, 10, or several decades. This approach requires the operator’s attention for marking the gain increments. We adopted a potential window, *E*_〈_*_j_*_〉_, as *x*, located around the *j*’s peak potential. The implementation of eq (1) with *G_j_*(*E*_〈_*_j_*_〉_), range programming (vs potential), can be provided by feedforward automatically, instead of by feedback.

The gain or range programming amplifier unit (fig. 1) comprises a comparator with variable threshold (Reference Voltage) and associated circuitry (Processing Ckt) for automatically changing the gain *G_j_*(*E*_〈_*_j_*_〉_) when the scanned electrode potential reaches a threshold of the *j*th potential window *E*_〈_*_j_*_〉_. The value of gain *G_j_* remains at the programmed value over the entire potential window *E*_〈_*_j_*_〉_. The processing circuit comprises a programmable gain amplifier, counter, and multiplexer/demultiplexer (MPX) and related circuitry for performing the proper functions. By comparing the sweep potential with the set voltage, the comparator causes the counter to produce a bit control signal to the demultiplexer, which selects a gain-determining feedback resistor, and, in turn, switches the next prespecified reference voltage to the comparator.

## Results and Discussion

**Figure 2:**
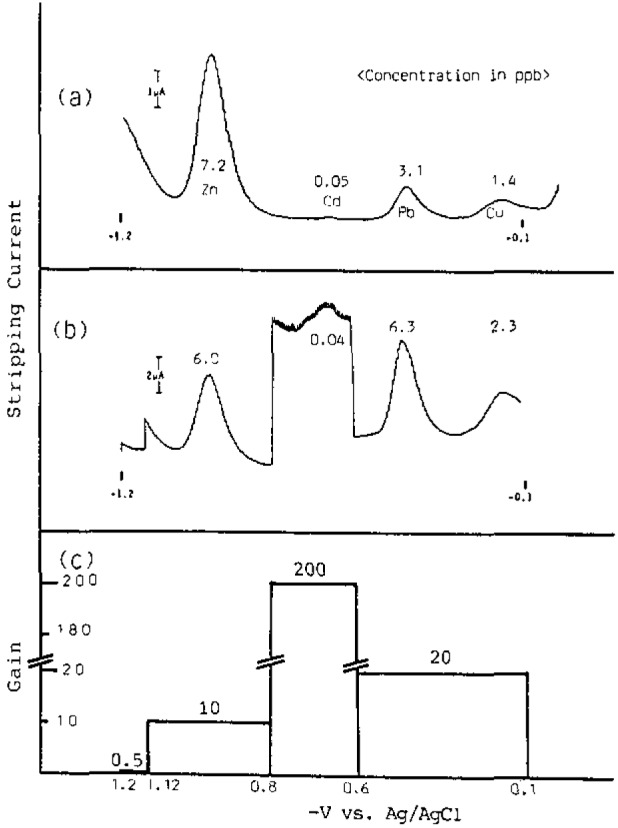
Typical voltammograms, (a) conventional, with deposition time of 30 min; (b) range programmed, with 3 min deposition with gain program shown in (c).

Figure 2 shows differential pulse stripping voltammograms with a range or gain program shown in (C) for a 10 mL aliquot of the filtered seawater sample solution (0.45 μm Millipore membrane) whose pH was adjusted to 3.0. The adverse concentration ratio (Zn/Cd) was 150. The readability, however, improved to the same order of magnitude in the range programming mode ((b) in fig. 2), without making any sacrifice in data quality. The potentiostat was a PAR Model 174A equipped with a Model 303 static mercury drop electrode.

**Figure 3:**
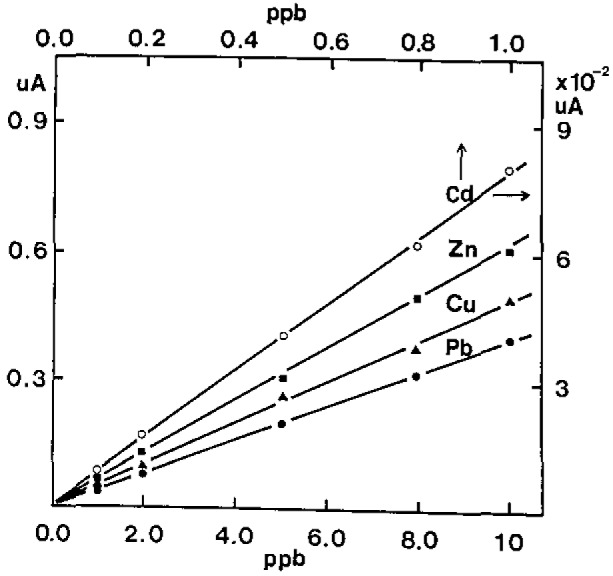
Standard addition calibrating curves in seawater. The peak currents are net values.

To establish linearity between peak current and concentration, standard curves for four metals in seawater were simultaneously run at pH 3.0. Standard additions were made by adding to the 10-mL sample, 10-μL volumes of a solution containing 0.100 ppm in Cd and 1.0 ppm in Zn, Pb, and Cu. The resulting standard addition curves (shown in fig. 3) demonstrate response linearity with good precision. With the deposition potential of −1.2 V (vs Ag/AgCl) and a deposition time of 3 min, we applied the technique to analyze simultaneously more than 300 seawater and marine samples employing the standard addition calibration curve and triple spiking procedure [7], respectively.
